# Electromagnetic Susceptibility Analysis of the Operational Amplifier to Conducted EMI Injected through the Power Supply Port

**DOI:** 10.3390/mi15010121

**Published:** 2024-01-11

**Authors:** Peng Huang, Bing Li, Mengyuan Wei, Xuchun Hao, Xi Chen, Xiaozong Huang, Wei Huang, Shuling Zhou, Xiaokang Wen, Shuguo Xie, Donglin Su

**Affiliations:** 1 School of Electronic and Information Engineering, Beihang University, Beijing 100191, China; penghuang@buaa.edu.cn (P.H.); xieshuguo@buaa.edu.cn (S.X.); 2 Research Institute for Frontier Science, Beihang University, Beijing 100191, China; 3 No. 24 Research Institute, China Electronics Technology Group Corporation, Chongqing 404100, China; hxzchn@163.com (X.H.);

**Keywords:** electromagnetic environmental effect, electromagnetic interference (EMI), electromagnetic susceptibility (EMS), operational amplifier (op-amp), susceptibility threshold, voltage follower

## Abstract

Operational amplifiers (op-amps) are widely used in circuit systems. The increasing complexity of the power supply network has led to the susceptibility of the power supply port to electromagnetic interference (EMI) in circuit systems. Therefore, it is necessary to investigate the electromagnetic susceptibility (EMS) of op-amps at the power supply port. In this paper, we assessed the effect of EMI on the operational performance of op-amps through the power supply port by a bulk current injection (BCI) method. Firstly, we conducted the continuous sine wave into the power supply port by a current injection probe and measured the change in the offset voltage under EMI. Secondly, we proposed a new method of conducted susceptibility and obtained the susceptibility threshold regularities of the op-amps at the power supply port under the interference of different waveform signals. Our study provided conclusive evidence that EMI reduced the reliability of the op-amp by affecting the offset voltage of op-amps and demonstrated that the sensitivity type of op-amps was peak-sensitive at the power supply port. This study contributed to a deep understanding of the EMS mechanism and guided the design of electromagnetic compatibility (EMC) of op-amps.

## 1. Introduction

**Figure 1:**
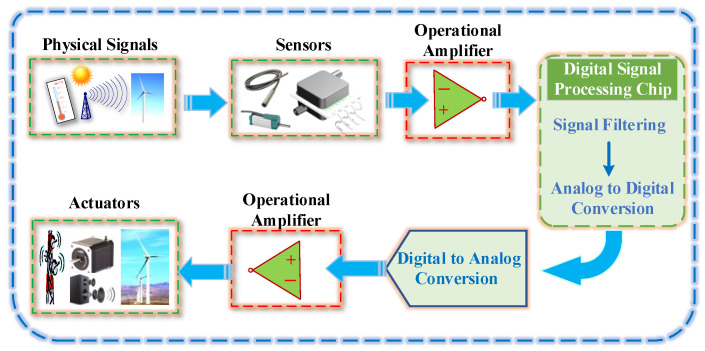
Signal transmission link system.

Recent advances in the field of integrated chip-manufacturing processes have fostered the ever-increasing need for the development of reliable and high-performance operational amplifiers (op-amps). Op-amps have become one of the most common electronic devices in the field of analog and control integrated circuits [1,2,3]. At the front end of the signal transmission link, the op-amp can sense and amplify the function of the input signal [4,5,6,7]. At the end of the signal transmission link, the op-amp enhances the drive capability of the system and performs impedance conversion. The architecture of the signal transmission link system is presented in Figure 1.

As chips become more integrated and data processing becomes faster, the electromagnetic interference (EMI) of chips must be methodically considered [8,9,10]. In addition, integrated circuits face more complex electromagnetic environments [3,11]. Op-amps as analog integrated chips are more susceptible to EMI [12,13]. The reliability of the op-amps directly affects the reliability of the whole circuit system. Therefore, some investigations have attracted more concern to reduce and improve the reliability of op-amps. For instance, 4H-SiC fabrication technology enables op-amps to operate at high temperatures with reliable performance [14,15]. An op-amp architecture that reduces the EMI effects has less EMI-induced offset [16,17,18]. Pseudo-differential inverters are commonly utilized to lessen the common-mode gain of the op-amps and enhance the differential mode gain to improve the common-mode rejection ratio of the op-amps [19]. The op-amp with symmetric topologies is capable of increasing immunity against EMI [20]. Due to the variety of interference sources in the electromagnetic environment, it is usually difficult to establish a very accurate electromagnetic environment model [21,22,23,24,25].

However, integrated circuits have many internal logic circuits and buffers. Their state switching can cause significant voltage drops and ripples in the distribution network, and supply voltage fluctuations can lead to severe EMC problems [26,27]. Prior research has neglected the impact of interference signal waveforms on the EMS properties of operational amplifiers. In addition, most people were concerned about the effect of injecting interference signals at differential inputs on op-amps, while injecting interference into power supply ports is lacking [28]. Furthermore, most research on power supply ports has only examined electrostatic discharges and low-frequency industrial-frequency signal interference, neglecting to address the impact of high-frequency signals on power supply ports. Moreover, the relationship between the electromagnetic susceptibility of op-amps and the type of interfering signals has yet to be investigated. We analyzed the effect of the type of interfering signal on the susceptibility threshold regularity of op-amps from several typical interfering signal waveforms.

In this paper, we focused on the EM characteristics of op-amps to conduct electromagnetic interference injected through the power supply port. Firstly, the continuous sine wave was injected into the power supply port, and the reliability of the op-amp reduced as the offset voltage increased significantly when the interference signal intensity exceeded the susceptibility threshold. Secondly, to obtain the comprehensive EMS of op-amps, five different waveforms were injected into the power supply port, respectively, and it revealed that the stability of the EMS threshold was essentially related only to the peak value and frequency of the interfering signal, not the duty cycle and bandwidth. The findings greatly contributed to the design of an electromagnetic protection of the op-amp.

The innovations of this paper can be summarized as four major aspects: (i) It was confirmed that EMI through the power port affected the op-amp’s performance parameters; (ii) In the conducted susceptibility tests, we employed several different interference signals from the interference in the two standards MIL-STD-461G and GJB 151B-2013 to investigate the electromagnetic susceptibility characteristics of the op-amps [29,30]; (iii) Electromagnetic susceptibility threshold regularities of op-amps were found under signal interference of different waveforms; (iv) We discovered that the op-amp’s susceptibility type was a peak type, and that the duty cycle and offset frequency of the modulated signal had little effect on the electromagnetic susceptibility thresholds of op-amps.

The rest of this article is structured as follows. In Section 2, we analyze the internal circuit characteristics of op-amps. Subsequently, we investigate the impact of EMI on the offset voltage of op-amps. Additionally, we conducted experiments to assess the susceptibility of op-amps to conducted EMI. In Section 3, we analyze the EMS characteristics under various waveform signals. In Section 4, we analyze the susceptibility and the difference between the proposed and the traditional test methods. Finally, we deliver the major conclusions.

## 2. Device and Experiment

### 2.1. Characteristics of Operational Amplifier Devices

**Figure 2:**
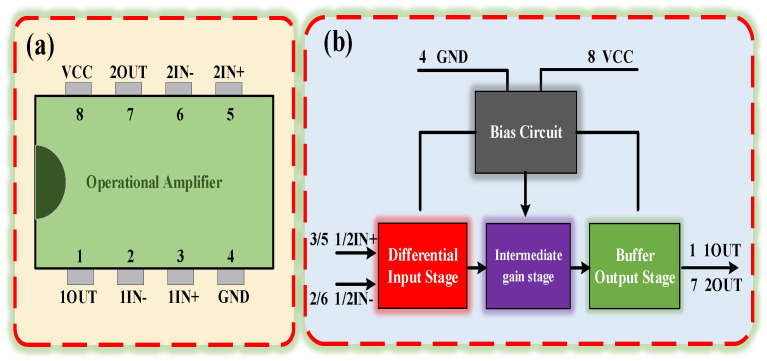
(**a**) Op-amp’s pin configuration and function. (**b**) The operational amplifier’s functional block diagram.

The op-amp model SF158MD was used to examine the EMS features of the op-amp. The op-amp was a monolithic integrated circuit fabricated using a bipolar process. Furthermore, the SF158MD chip internally consisted of two independently operating low-power op-amps. Considering that the dual op-amps inside the SF158MD chip were manufactured using the same craft, we believed that the dual op-amps had the same susceptibility characteristics, so we selected one of the op-amps as the object of investigation. Figure 2a depicts this op-amp’s pin arrangement and function. The op-amp’s internal circuit consisted of four main circuit modules: differential input stage, intermediate gain stage, buffer output stage, and bias circuit. The op-amp’s functional block diagram is shown in Figure 2b.

**Figure 3:**
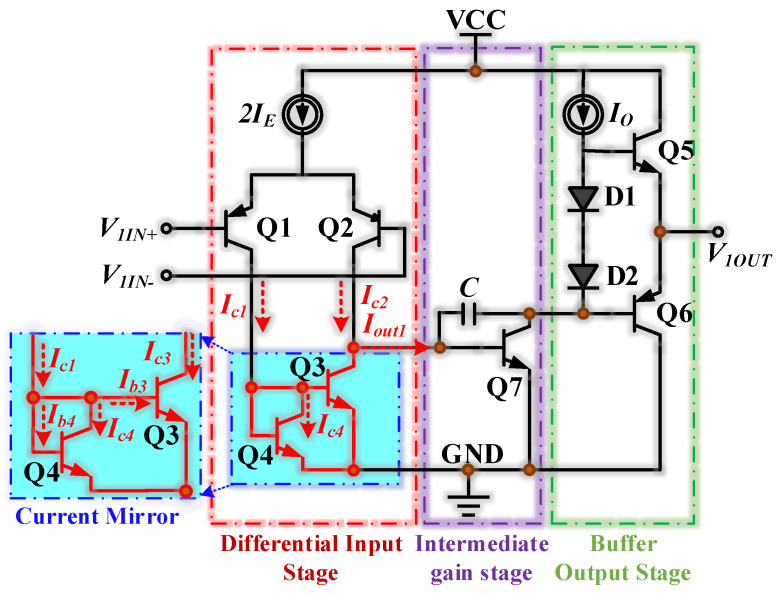
The simplified op-amp circuit diagram.

The SF158MD we used is the same type of operation amplifier as the LM158, LM358B, and LM358BA devices. By referencing the chip manuals, we found they had the same functional structure with similar differential input circuits, current mirror circuits, intermediate stage amplifier circuits, bias circuits, and buffer output circuits. Therefore, we considered that their simplified op-amp circuits are similar. Figure 3 shows the simplified op-amp circuit diagram. The purpose of the current mirror in the input stage was to supply an arbitrary DC bias current to the integrated circuit (IC). Simultaneously, the current mirror ensured that the output current *I_out_*_1_ was constant. The function of the differential input stage was to amplify differential signals and convert them into single-ended signals. The formula for calculating the PN junction currents of the two transistors passing through the current mirror is as follows.

(1) $$\left\{ \begin{array}{l} I_{c3}=I_{s3}\left(e\frac{V_{be3}}{V_{T}}-1\right)\\ I_{c4}=I_{s4}\left(e\frac{V_{be4}}{V_{T}}-1\right)\\ V_{T}=\frac{KT}{q} \end{array}\right.$$

In the differential input stage, two pairs of matched transistors, Q1–Q2 and Q3–Q4, constituted a symmetrical circuit structure. *I_c_*_1_, *I_c_*_2_, *I_c_*_3_, and *I_c_*_4_ were the collector currents of the differential input stage transistors Q1, Q2, Q3, and Q4, respectively. *I_b_*_3_ and *I_b_*_4_ corresponded to the base currents of Q3 and Q4, respectively. Moreover, transistors Q3 and Q4 constituted a current mirror. A transistor had two PN junctions, of which *I_s_* was the reverse saturation current of the PN junctions, *V_be_* was the applied voltage across the PN junction, *V_T_* was the voltage equivalent of the temperature, *K* was Boltzmann’s constant, *T* was the temperature in Kelvin scale, and *q* was the charge of the electron. Since Q3 and Q4 were matched, *V_be_*_3_ = *V_be_*_4,_ and *I_s_*_3_ = *I_s_*_4_. As shown below, we can obtain the relationship between the two currents, *I_c_*_3_ and *I_c_*_4_.

(2) $$I_{c3}=I_{c4}$$

We may construct the relationship equation for *I_c_*_1_, *I_b_*_4_, *I_b_*_3_, and *I_b_*_4_ using Kirchhoff’s Current Law (KCL), as shown below.

(3) $$\left\{ \begin{array}{l} I_{c1}=I_{b3}{+I}_{b4}+I_{c4}\\ I_{b3}=\frac{I_{c3}}{\beta_{3}}\\ I_{b4}=\frac{I_{c4}}{\beta_{4}} \end{array}\right.$$

*β*_3_ and *β*_4_ were the current amplification factors of transistors Q3 and Q4, respectively. Under the assumption that these two triodes were matched, *β*_3_ and *β*_4_ were the equal *β*_3_ = *β*_4_ = *β*. Formula (4) can be derived from Formulas (2) and (3).

(4) $$I_{c1}=I_{c4}(\frac{2}{\beta }+1)$$

If *β* >> 2, we can obtain *I_c_*_1_ = *I_c_*_3_ = *I_c_*_4_. This ensures that the current mirror provides a consistent output current *I_out_*_1_, which keeps the op-amp operating at a stable performance.

### 2.2. Op-Amp Offset Voltage Test under EMI

**Figure 4:**
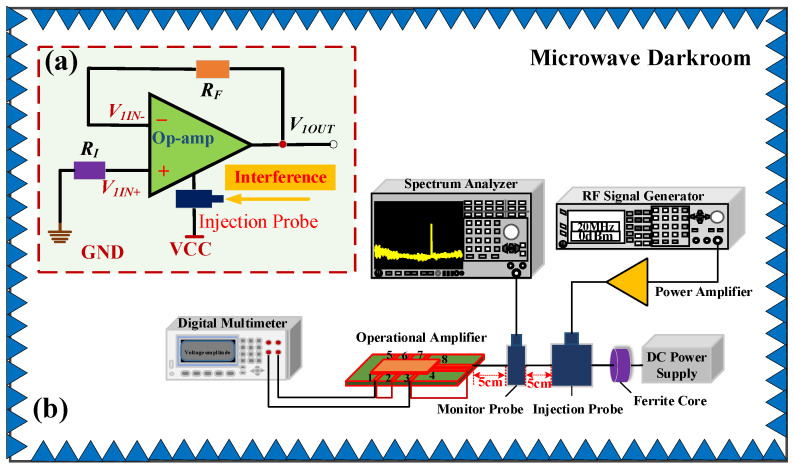
(**a**) Op-amp’s offset voltage test circuit under EMI. (**b**) Block diagram of the op-amp’s offset voltage test.

The offset voltage was a crucial op-amp performance indicator of the op-amp and directly determined the operating state of the op-amp. The voltage follower circuit was chosen to analyze the effect of EMI at the power supply port on the op-amp’s offset voltage. The op-amp of the voltage follower acted as a buffer and did not amplify the signal. Moreover, the output voltage of the voltage follower was the same as the input voltage. The influence of EMI on op-amps was more easily detected by a voltage follower composed of op-amps. The op-amp’s offset voltage test circuit under EMI is shown in Figure 4a. We connected the non-inverting input port of the op-amp to the ground while monitoring the input voltage values *V*_1*IN+*_ and the output voltage values *V*_1*OUT*_. Under ideal conditions, the input and output voltages of the follower were both 0 V. The inverting input of the test circuit was directly connected to the output port. *R_F_* and *R_I_* represented the internal impedances of the wiring, which were small enough to be ignored. The block diagram of the op-amp offset voltage test is shown in Figure 4b. The experimental block diagram comprised the following components: digital multimeter, spectrum analyzer, radio frequency (RF) signal generator, RF power amplifier, DC power supply, ferrite core, bulk current injection probe, monitor probe, SF158MD op-amp, and waveform generator.

### 2.3. Op-Amp Conducted Electromagnetic Susceptibility Test

**Figure 5:**
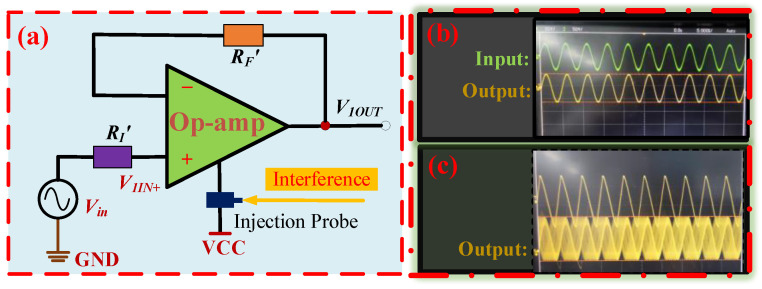
(**a**) Test circuit schematic for op amp in working condition under EMI. (**b**) The input and output waveforms of the op-amp under normal operating conditions. (**c**) The output waveform of the op-amp under abnormal operating conditions caused by EMI.

To investigate the EMS characteristics of op-amps during operation, we devised a voltage follower circuit to analyze the op-amp’s electromagnetic susceptibility (EMS). The circuit schematic is depicted in Figure 5a. The input signal *V_in_*, produced by the waveform generator was used as the operational input signal for the op-amp. The inverting input of the test circuit was directly connected to the output port. *R_F_*’ and *R_I_*’ represented the internal impedances of the wiring, and the resistance value was small and negligible. The interference signals were injected into the op-amp’s power supply port through the injection probe. As long as the interference strength was below the EMS threshold, the op-amp would remain functioning correctly, and the input signal’s voltage waveform would be identical to the output signal’s voltage waveform, as depicted in Figure 5b. Conversely, if the intensity of the interference surpassed the threshold of EMS, it would result in the op-amp functioning abnormally. The op-amp’s abnormal operation increased the peak-to-peak voltage waveform of the output signal, which differed significantly from the voltage waveform of the input signal, as depicted in Figure 5c. The peak-to-peak voltage of the input and output waveforms of the op-amp was 1.5 V when there was no EMI. We stipulated that when the peak-to-peak voltage of the output waveform fluctuated up and down by more than 0.5 V, the op-amp was considered to be in an electromagnetic susceptibility. It meant that when the op-amp was electromagnetically susceptible, the peak-to-peak voltage of the output waveform of the op-amp was more than 2 V or less than 1 V. Figure 5c shows the output waveform of the op-amp under abnormal operating conditions caused by EMI. Under the influence of electromagnetic interference, the output waveform jitter changed and was no longer the same as the waveform in Figure 5b, and the peak-to-peak voltage of the output signal waveform greatly exceeded the proposed 2 V. At this point, the op-amp was in an electromagnetically sensitive state.

In the op-amp EMS test, several different signal waveforms from the RF source were injected into the power line of the op-amp by a bulk current injection probe. When the interfering signal’s strength increased, the output waveform’s peak-to-peak voltage exceeded 0.2 times the peak-to-peak voltage of the input signal. This indicated that the interfering signal achieved the susceptibility threshold of the operational amplifier at the frequency point. The conduction susceptibility tests utilized single-frequency continuous wave signals, pulse-modulated (PM) signals, and frequency-modulation (FM) signals as the interference signals for the op-amp. Table 1 displays the various types and characteristics of the interference signals. The continuous wave was a sinusoidal signal that spanned from 10 kHz to 400 MHz, representing an analog signal. Pulse-modulated signals exhibited varying duty cycles and a pulse frequency of 1 kHz, which differed from the standards presented in IEC 62132, MIL-STD-461G, and GJB 151B-2013 standards. Our test methods were based on all three of these test standards. The FM signals were sinusoidal signals modulated with variable frequency offsets. The fundamental waveform was a 10 kHz sinusoidal signal, which represented conventional linear FM radar signals. The carrier frequency range of pulse-modulated and FM signals was from 10 kHz to 400 MHz.

**Figure 6:**
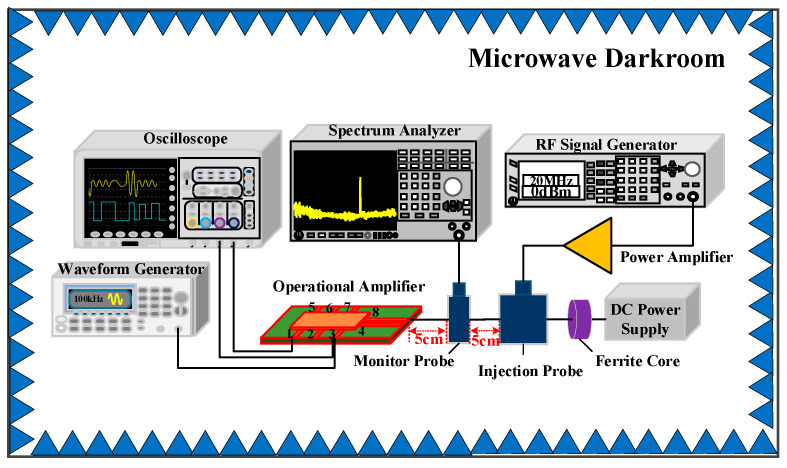
Block diagram of electromagnetic conducted susceptibility test of the op-amp.

**Figure 7:**
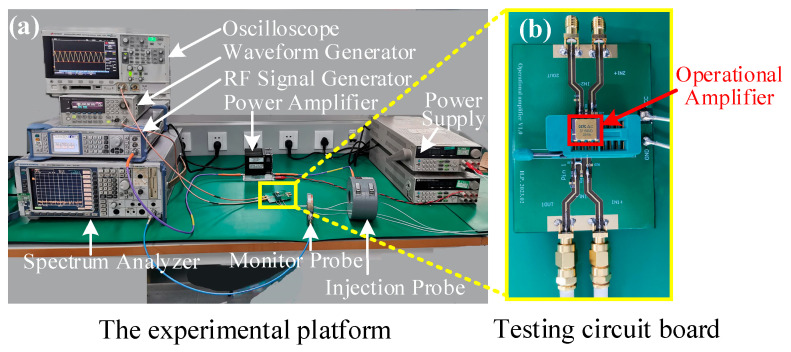
(**a**) Experimental platform for electromagnetic conduction susceptibility of the operational amplifier. (**b**) Testing circuit board.

The block diagram of the EMS test that was performed on the operational amplifier in this investigation is shown in Figure 6. The experimental block diagram comprised the following components: oscilloscope, spectrum analyzer, radio frequency (RF) signal generator, RF power amplifier, DC power supply, ferrite core, bulk current injection probe, monitor probe, SF158MD operational amplifier, and waveform generator. The waveform generator supplied a sinusoidal signal with a frequency of 200 kHz and a peak-to-peak voltage of 1 V to the input port of the op-amp as its functional signal. The oscilloscope observed the input signal waveform and output signal waveform of the op-amp in real-time. The RF signal generator generated various interference signals, which were injected into the operational amplifier’s power supply wires by a bulk current injection probe. Both the injection probes and monitoring probes we used work efficiently in the 10 kHz–400 MHz band. Concurrently, the strength of the interference signal linked to the op-amp supply wire was monitored using a spectrum analyzer and a current monitoring probe. The DC power supply supplied 15 V to the op-amp. The ferrite core was shielded against EMI for the DC power supply. The operational amplifier’s electromagnetic susceptibility test platform and the experimental instrumentation’s connection are shown in Figure 7a, and the test circuit board is depicted in Figure 7b.

The EMS test of the op-amp was conducted in a microwave darkroom to prevent electromagnetic interference from the external environment and maintain the reliability of the experimental process. The op-amp’s EMS test’s preparation stage referred to MIL-STD-461G and GJB 151B-2013. The EMS test procedure for op-amps was as follows.

(1) Preparation of the chip under test, the measuring instrument and the cables, and connection of the op-amp to the measuring device according to Figure 6. The current monitoring probe was placed 5 cm from the op-amp power supply port. The bulk current injection probe was positioned at a distance of 5 cm from the monitoring probe.
(2) Configuration of the test platform: Microwave-absorbing materials were placed around the test platform to reduce the impact of space electromagnetic fields on the test and ensure the experiment’s reliability. We arranged the site of the experiment in a microwave darkroom. The power supply could provide a stable power supply to the operational amplifier and experimental equipment to ensure accurate measurements of the operational amplifier and the measuring instrument.
(3) Calibration of the measuring equipment: We calibrated the bulk-current injection probes, proper functioning of the operational amplifiers, and signal generator. The signal generator was capable of providing several proposed interference signals.
(4) The susceptibility test of op-amps: Firstly, we increased the intensity of the interference signal gradually by the bulk current injection probe until the op-amp appeared to be a susceptible phenomenon. Secondly, we recorded the susceptibility phenomenon and the input power of the interference signal.
(5) Experimental data processing and susceptibility analysis: We recorded the strength of the interference signal during susceptibility testing, established susceptibility threshold curves, and investigated its EMS properties.

## 3. Results

### 3.1. Offset Voltage Test Results

**Figure 8:**
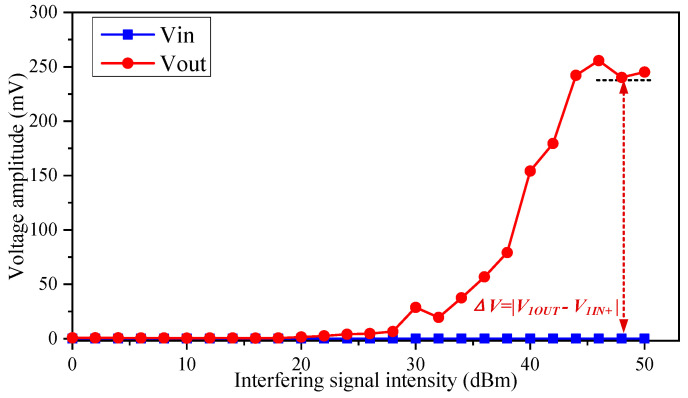
The effect of interfering signal intensity on offset voltage.

The voltage values of the input and output ports of the voltage follower were set as 0.02 mV and 0.6 mV, respectively, in the absence of the EMI signal. The voltage Δ*V* = |*V*_1*out*_ − *V*_1*IN+*_| was the output offset voltage of the op-amp. Figure 8 depicts the EMI effect on the offset voltage of the op-amp. In the experiments, a 380 MHz interference signal was coupled to the connecting cable of the op-amp power supply port through the injection probe. When the interfering signal intensity was less than 28 dBm, the input and output signal voltages and the op-amp offset voltage were near 0 V. However, when the intensity of the interference signal exceeded 28 dBm, the offset voltage was substantially raised, which reduced the reliability of the op-amp. An op-amp with the EMI could produce an offset voltage of more than 200 mV, which was significantly different from normal operating conditions. The work confirmed that EMI at the op-amp’s power port affects its performance parameters.

### 3.2. Conducted Susceptibility Test Results

**Figure 9:**
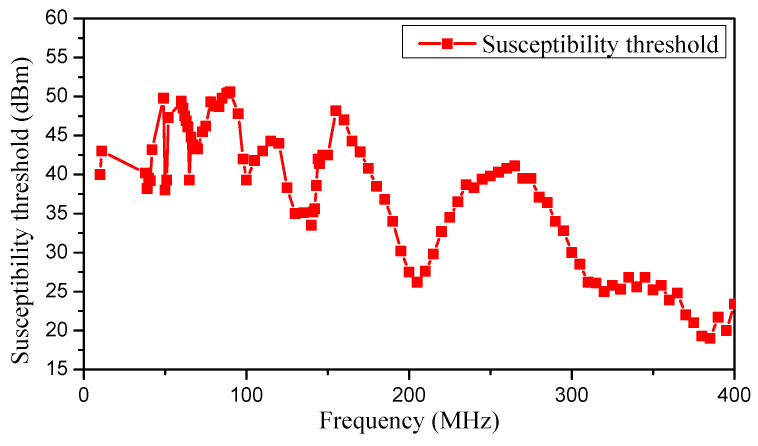
The EMS threshold curve of the op-amp subjected sinusoidal signal interference.

A continuous sine wave with a frequency of from 10 kHz to 400 MHz was injected into the power port of the op-amp as an interference signal. There were differences in susceptibility thresholds at different frequency points. The op-amp was hardly susceptible to it when injecting interfering signals with frequencies below 10 MHz. When the frequency of the interfering signal was around 230 MHz and in the high-frequency band from 310 MHz to 400 MHz, it was more likely to make the op-amp electromagnetically susceptible. Under the current test conditions, the op-amp was not electromagnetic susceptible when the frequency of the interfering signal was lower than 10 MHz, so the susceptibility thresholds in the range from 10 kHz to 10 MHz were not shown on the threshold curves. On the contrary, the frequency bands in which the op-amps became electromagnetically susceptible were able to be shown on the threshold curves. The EMS threshold curve of the op-amp in the presence of sinusoidal signal interference is demonstrated in Figure 9.

**Figure 10:**
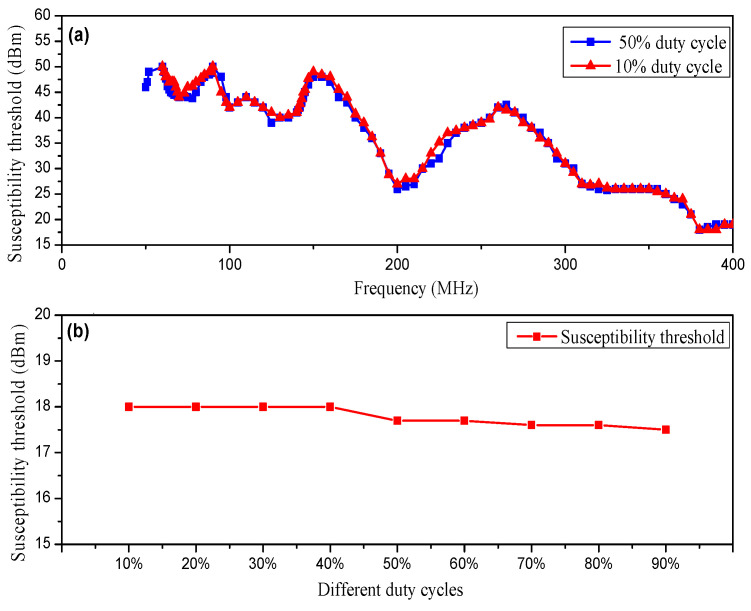
(**a**) The EMS threshold curves of the op-amps under pulse-modulated interference signals with various duty cycles. (**b**) EMS threshold curve of the op-amp under pulse-modulated interference signals with various duty cycles at 380 MHz.

The pulse-modulated signals with a pulse period of 1 ms, and duty cycles of 10% and 50% were considered interference signals. Figure 10 illustrates the electromagnetic susceptibility threshold from 10 kHz to 400 MHz. The op-amp was hardly susceptible to it when the frequency of interfering signals was below 50 MHz. The EMS threshold curves subjected to pulse modulation (PM) signal interference were similar to the previously mentioned continuous sine wave signal interference. All of them had lower susceptibility thresholds near the 200 MHz frequency point and within the frequency range from 310 MHz to 400 MHz. The EMS threshold curves of the op-amp under pulse-modulated interference signals with different duty cycles are presented in Figure 10a. The sinusoidal continuous wave signal was regarded as the basic interference signal, and the single frequency point was selected based on the susceptibility threshold curve when the sinusoidal continuous wave was used as the interference signal. The minimum value of the electromagnetic susceptibility threshold for sinusoidal signal interference received by the op-amp corresponded to a frequency point of 380 MHz, which means that this frequency point was the most susceptible to the effects of EMI. Therefore, we chose this particular 380 MHz frequency point to analyze the electromagnetic susceptibility characteristics of the op-amp.

We performed EMS tests at a fixed carrier frequency of 380 MHz and selected pulse-modulated signals with a duty cycle of from 10% to 90% in 10% steps as the interference signals. According to the experimental results, the EMS thresholds of the op-amp remained constant under the interference of pulse-modulated signals with various duty cycles. The EMS threshold curve of the op-amp under pulse-modulated interference signals with different duty cycles at 380 MHz is presented in Figure 10b.

**Figure 11:**
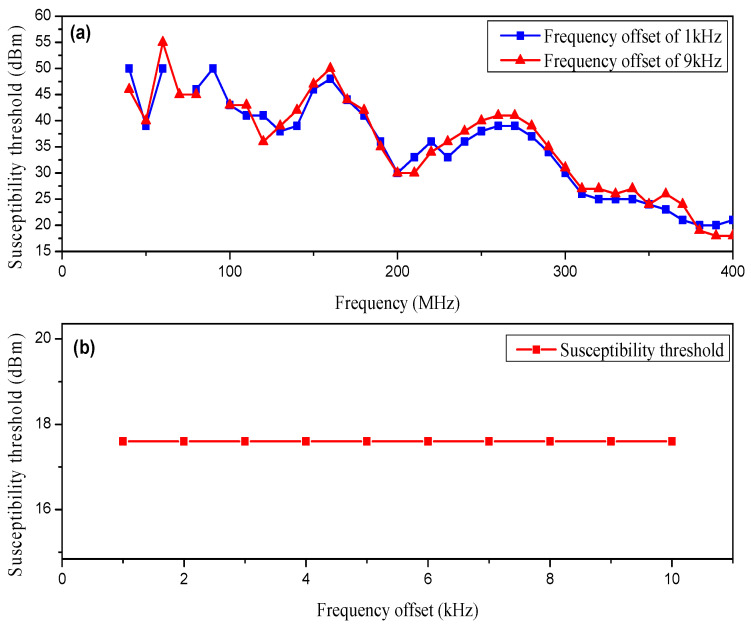
(**a**) The EMS threshold curves of the op-amp due to interference by sinusoidal frequency modulation signal interference with various frequency offsets. (**b**) EMS threshold curve of the op-amp due to interference by sinusoidal frequency modulation signal interference with different frequency offsets at 380 MHz.

Figure 11a shows the EMS threshold curves of the op-amp under the interference of sinusoidal frequency modulation (FM) signals with a frequency offset of 1 kHz and 9 kHz, respectively. The op-amps were hardly susceptible to it when the frequency of interfering signals was below 40 MHz. The carrier frequencies of interference signals had harmonic components at 40 MHz, 80 MHz, 120 MHz, and 200 MHz. The threshold of op-amps had less susceptibility near these frequency points and was more susceptible to EMI. Figure 11b illustrates the EMS threshold curve of an op-amp when the sinusoidal FM signal with different frequency offsets and a fixed carrier frequency of 380 MHz were the interfering signals. The susceptibility threshold of the op-amp remained essentially constant under the interference of sinusoidal FM signals with different frequency offsets.

**Figure 12:**
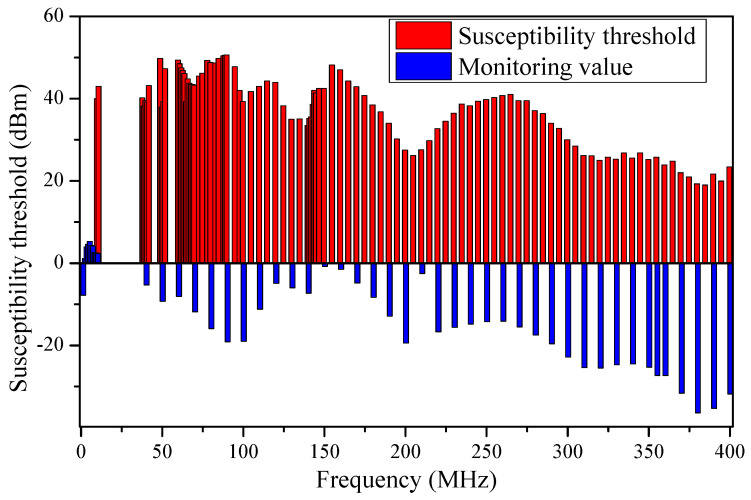
The EMS threshold thresholds in the presence of a single-frequency interference and monitoring values of monitoring probes.

Figure 12 shows that the monitored value measured by the monitor probe reflected the amount of energy coupled to the cable by the actual interference signal. The monitoring power can reflect the frequency selection characteristics of the op-amp, and the interference signal intensity of the RF source output can also reflect the susceptibility at different frequencies. The monitoring power and the output power of the RF source all reflect the electromagnetic susceptibility characteristics of the op-amp, so the output signal intensity of the RF source is chosen as the susceptibility threshold. We found that the monitoring values in the low-frequency band (<80 MHz) were almost all larger than −10 dBm, while the monitoring values in the high-frequency band (>230 MHz) were less than −20 dBm. Based on the comparison of the values of the monitoring probe, the interference signal in the low-frequency band may be coupled to the power supply cable of the op-amp more efficiently. However, the interference signal in the high-frequency band was more difficult to couple to the power supply cable of the op-amp. At the same time, we found that the op-amp had a higher susceptibility threshold at low frequencies and a lower susceptibility threshold at high-frequency bands. Although it was challenging for high-frequency interference signals to be coupled to the power supply cable of the op-amp, the op-amp was more susceptible to electromagnetic susceptibility from high-frequency interference signals, which could compromise their reliability.

## 4. Discussion

### 4.1. Susceptibility Analysis

As seen in the results in Section 3, we obtained the EMS threshold regularities for op-amp under interference signals with varying waveform parameters, such as frequency, modulation types, duty cycles, and frequency offsets. According to the experimental data in Figure 8, Figure 9 and Figure 10, the op-amp had lower susceptibility thresholds to the high-frequency band signals, which meant that the op-amp was more easily affected by the high-frequency bands’ interference. However, the interfering signals in the high-frequency band were more difficult to couple to the cable compared to the signals in the low-frequency bands in Figure 12. The reason why the op-amp operated abnormally was that the EMI signals coupled into the op-amp through the power supply cable and generated a DC shift [31]. EMI led to a non-linear reaction in the diode formed by the silicon PN junction in the op-amp. The non-linear response resulted in the production of a DC shift, as the interfering signals were rectified and converted into a DC signal.

**Figure 13:**
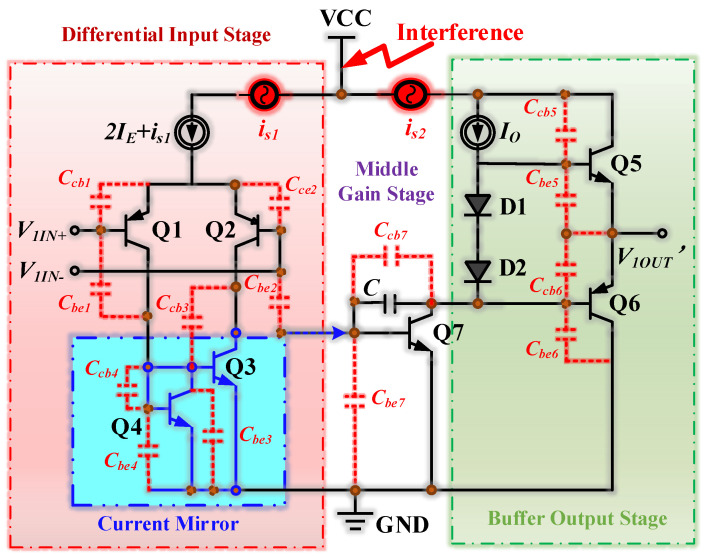
The simplified op-amp circuit diagram under EMI.

Moreover, if the frequency of the high-frequency interference signals was much higher than the op-amp’s bandwidth, the parasitic capacitances between the PN junction of the transistors inside the op-amp must be considered. The simplified op-amp circuit diagram under EMI is shown in Figure 13. *i_s_*_1_ and *i_s_*_2_ represented the currents coupled into the op-amp by the interfering signal. {(*C_cb_*_1_, *C_be_*_1_), (*C_cb_*_2_, *C_be_*_2_), (*C_cb_*_3_, *C_be_*_3_), (*C_cb_*_4_, *C_be_*_4_), (*C_cb_*_5_, *C_be_*_5_), (*C_cb_*_6_, *C_be_*_6_), (*C_cb_*_7_, *C_be_*_7_)} represented the parasitic capacitance between the two PN junctions of {Q1, Q2, Q3, Q4, Q5, Q6, Q7} transistors, respectively. The parasitic capacitance disrupted the symmetrical balance of the differential amplification circuit, resulting in an unstable output from the current mirror. This instability ultimately produced a DC shift in the op-amp, negatively impacting its performance.

### 4.2. Differences in Test Methods

Within an intricate electromagnetic setting, all disruptive signals would ultimately impact the operational amplifier through the printed circuit board (PCB) traces or wire conduction, despite the electromagnetic interference being sent or emitted into the circuit system. Consequently, in this work, we proposed a novel BCI measurement methodology to investigate the susceptibility properties of the op-amp power supply port. According to IEC Standard 62132, various conventional methods for integrated chips mainly include direct power injection (DPI), a transverse electromagnetic (TEM) cell, a gigahertz transverse electromagnetic (GTEM) cell, and bulk current injection (BCI) [32,33]. TEM and GTEM are both radiated emission immunity tests, and they do not accurately analyze the EMS of the power supply ports of op-amps. BCI and DPI are both conducted emission immunity measurement methods. The DPI test method required additional design and processing of the test board, which was expensive. Moreover, the DPI test method cannot conduct tests in the chip’s actual working environment. On the contrary, the BCI test method can directly carry out conducted susceptibility tests in the actual working environment of the chip without the need to design and process PCBs. Moreover, the BCI test was easy to operate. Standard BCI tests use a single pulse-modulated signal as the interference signal, so they cannot check how sensitive op-amps are to different electromagnetic environments. Compared to the traditional BCI method, the suggested method can obtain the electromagnetic susceptibility profiles of the op-amp in more dimensions by using signals with different waveforms as interference signals. There were no specialized cable ducts to fix the cables and to keep the cables 5 cm above the reference plane at the time of the test conditions. Throughout the test, to ensure that the test conditions were as consistent as possible, we did not stop the test and did not move the cables. However, it was hard to achieve repeatable susceptibility thresholds if repeat tests were conducted again after removing the test conditions. Because of the change in cable placement, the loop impedance was affected. This was also a shortcoming of this article. However, instead of focusing excessively on the frequency points of susceptibility and the magnitude of the susceptibility threshold, we paid more attention to the relationship between the waveform characteristics of different interfering signals and the susceptibility threshold curves. Therefore, this shortcoming had little effect on our investigation.

## 5. Conclusions

In this paper, we investigated the susceptibility properties of an op-amp through the power supply port. We injected interference signals by conduction at the power supply port to study the effects of EMI on the offset voltage. Moreover, we injected several different waveforms of interference signals into the power supply cables to investigate the effects of different waveforms on the electromagnetic susceptibility thresholds of the op-amp. We had shown that the op-amp’s offset voltage changed dramatically when the interfering signal intensity exceeded 28 dBm. We found that the op-amp was not susceptible when the interfering signal frequency was less than 50 MHz. However, in the high-frequency band of from 300 MHz to 400 MHz, the susceptibility thresholds of the op-amps were all below 25 dBm and were more susceptible to EMS than in the low-frequency band. Moreover, the results revealed that the EMS of the op-amp was a type of “peak” susceptibility. The stability of the EMS threshold of the op-amp was essentially related only to the peak value and frequency of the interfering signal, not the duty cycle and bandwidth. This approach could obtain the EMS boundary of op-amps more comprehensively, which provides an effective method for evaluating the EMS characteristics of the op-amp. In addition, the comprehensive EMS boundary provides theoretical support for the electromagnetic protection of the circuit system.

## Figures and Tables

**Table 1:** Types and characteristics of the interference signals.

Interference Signal Types	Interference Signal Characterizations
Continuous wave	Continuous sine wave Frequency band: 10 kHz–400 MHz
Pulse-modulated signals	Duty cycles:10–90% Carrier frequency range: 10 kHz–400 MHz Fundamental waveform: pulse signals
Frequency-modulation signals	Frequency offsets: 1 kHz–10 kHz Carrier frequency range: 10 kHz–400 MHz Fundamental waveform: sinusoidal signals

## Data Availability

All the data are contained within this article.
